# Disappearance and dissemination of sleep symptoms: the importance of sleep medicine expertise for psychiatry. A comment on Forbes *et al*.

**DOI:** 10.1017/S0033291724001831

**Published:** 2024-09

**Authors:** Vincent P. Martin, Régis Lopez, Jean-Arthur Micoulaud-Franchi, Christophe Gauld

**Affiliations:** 1 Deep Digital Phenotyping unit, Department of Precision Health, Luxembourg Institute of Health, L-1445 Strassen, Luxembourg; 2 National Reference Centre for Orphan Diseases, Narcolepsy-Rare Hypersomnia, Sleep Unit, Department of Neurology, CHU de Montpellier, University of Montpellier, Montpellier, France; 3 Institute for Neurosciences of Montpellier (INM), University of Montpellier, Inserm, Montpellier, France; 4 Univ. Bordeaux, CNRS, SANPSY, UMR 6033, F-33000 Bordeaux, France; 5 University Sleep Clinic, University Hospital of Bordeaux, Place Amélie Raba-Leon, 33 076 Bordeaux, France; 6 Service de Psychopathologie du Développement de l'Enfant et de l'Adolescent, Hospices Civils de Lyon & Université de Lyon 1, Lyon, France

**Keywords:** classification, DSM-5, sleep medicine, symptom delineation

## Abstract

Commentary of ‘Elemental psychopathology: distilling constituent symptoms and patterns of repetition in the diagnostic criteria of the DSM-5’ Vincent P. Martin 1, Régis Lopez 2,3, Jean-Arthur Micoulaud-Franchi 4,5, Christophe Gauld 4,6,*

To the Editors,

We read with great interest the article by Forbes et al. (2024), in which the authors map the symptoms extracted from the 202 diagnoses of adult mental disorders in Section II of the DSM-5 to quantify symptom overlap between diagnoses. This major work required a substantial research effort since the authors manually inventoried the 628 symptoms of the DSM-5. We meticulously examined Chapter 12 on Sleep-Wake Disorders. Indeed, sleep disorders and other mental disorders are known to be closely interwoven (Freeman, Sheaves, Waite, Harvey, & Harrison, 2020). Gauld et al. (2021b) already extracted the different symptoms of chapter 12 of the DSM-5 in a previous work. However, the two extractions by Gauld and Forbes do not align. Moreover, sleep disorders are also classified in a dedicated disorder classification, the International Classification of Sleep Disorders (*ICSD-3*, 2014), for which Gauld et al., also did a symptom extraction (Gauld et al., 2021a). The goal of this commentary is to identify, explain and discuss the disagreements between extractions of sleep symptoms from the DSM-5 (by Forbes and by Gauld) and from the ICSD-3 (by Gauld). The alignment between the three symptom extractions is available online^1^ and in Fig. 1.

**Figure 1.**
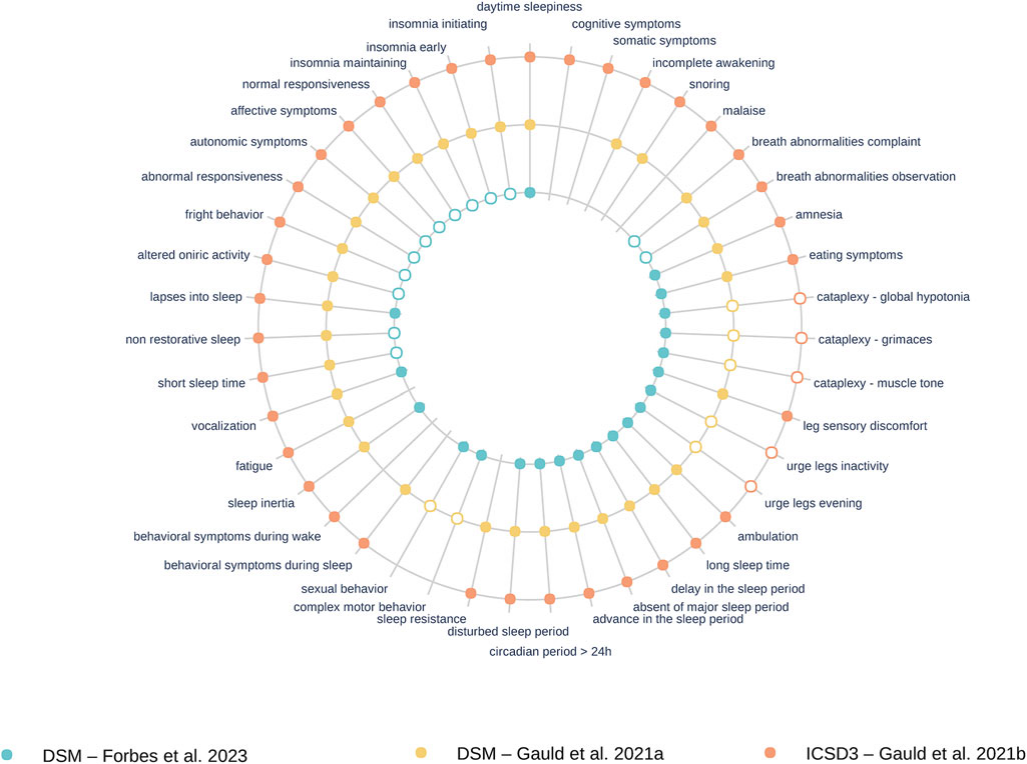
Symptom repetition in the DSM-5 Sleep-Wake disorder chapter in the studies of Forbes et al. (2024) and Gauld et al. (2021b), and in the ICSD-3 by Gauld et al. (2021a). The rings within each circle correspond to different analyses, and each dot is a symptom. Filled dots are extracted symptoms with the same granularity in all the extractions; empty dots represent lumped symptoms.

We discuss the clinical implications of four different alignments within the context of lumping *v.* splitting (American Psychiatric Association, 2013, p. 361). Splitting refers to dividing a category into two or more categories, while lumping involves combining two or more distinct categories into a single category.

We first discuss lumps and splits with regard to clinical diagnoses. A striking example is the extraction of insomnia as a unique symptom in insomnia disorder and circadian disorders by Forbes et al., [S301], whereas Gauld et al., differentiate between three very classical (in the field of sleep medicine) types of insomnia symptoms: ‘Difficulty initiating sleep’, ‘Difficulty maintaining sleep’ and ‘Waking up earlier than desired’. This is not solely a linguistic refinement: such differentiation makes it possible to envisage different diagnoses and treatment plans. Conversely, regarding cataplexy in the B1 criterion for narcolepsy, Gauld et al., extract only one symptom, while Forbes et al., differentiate three symptoms depending on the motor manifestations of cataplexy [S128, S129, and S130]. However, from a sleep medicine perspective, this split is not relevant: according to narcolepsy experts, the relevant differentiation is more a question of the certainness and typicality of cataplexy than its motor manifestations (Kornum et al., 2017).

Some lumps performed by Forbes et al., potentially lead to clinical inaccuracy, such as the implicit lump between fatigue and hypersomnia in the analysis of obstructive sleep apnea syndrome (OSAS, criterion A1b). Indeed, while sleepiness and fatigue overlap in everyday vocabulary (Hirshkowitz, 2013), they have significant diagnostic and therapeutic implications (Shen, Barbera, & Shapiro, 2006). A similar outcome concerns the lumping of poor sleep quality (qualitative) and short sleep time (quantitative) by Forbes et al., into a single symptom [S567: ‘Dissatisfaction with sleep quantity or quality’]. They thus attribute the same lumped symptom to insomnia disorder (criterion A) and OSAS (criterion A1b), whereas short sleep time is not a diagnostic criterion for the latter (Lévy et al., 2015).

Forbes et al., differentiate two dream-related symptoms [S425: ‘No or little dream imagery is recalled’] from NREM Sleep Arousal Disorder (SAD) criteria and [S139: ‘Extended, extremely dysphoric, and well-remembered dreams’] from Nightmare disorder, whereas Gauld et al., lump and rename them into a unique symptom: ‘Altered oneiric activity’. Similarly, Forbes et al., differentiate the ‘Sleep-related sexual behavior’ [S138, SAD] and the ‘complex motor behaviors’ criterion [S639, RBD] whereas Gauld et al., lump and rename them into a unique ‘Behavioral symptoms during night’. Although these lumps obscure important differences, they ensure that these symptoms, which are often presented by patients with SAD, RBD, or Nightmare disorder, encourage clinicians to carefully and rigorously search for specific symptoms of each of these disorders in order to distinguish them, which is the role of bridge symptoms (Castro et al., 2019). On the other hand, Forbes et al., lump all the symptoms related to criterion A2 of the SAD in the DSM: ‘Abrupt terror arousals from sleep with intense fear, signs of autonomic arousal, and relative unresponsiveness to comforting’ [S136]. Using this criterion, Gauld et al., extract four different symptoms: ‘Fright behavior’, ‘Affective symptoms’, ‘Autonomic symptoms’, and ‘Abnormal responsiveness’, which bridge the three parasomnia-related disorders. Again, this apparent similarity in the manifestations of such different disorders is a strong clue when searching for specific symptoms to differentiate them.

Finally, some differences revolve around the definition of what constitutes a symptom. For instance, Forbes et al., distinguish ‘Urge to move legs begins or worsens during periods of rest or inactivity’ [S426, A2 criterion, Restless legs syndrome] from ‘Urge to move legs is worse in the evening’ [S427, A3 criterion, RLS]. Gauld et al., lump them into a single symptom ‘Urge to move legs’ symptom. Indeed, the moment of the day during which the symptom occurs specifies the context, not the symptom. Moreover, the clinical focus of Gauld et al., led them to identify ‘insomnia’ symptoms within criterion B of Nightmare disorder, criterion C of RBD and criterion A3 of Restless legs syndrome, whereas Forbes et al., do not extract them. Since these three disorders share the common complaint of wakening or awakening, they have been identified as insomnia symptoms from a clinical point of view.

Whereas the extraction by Forbes et al. (2024) aims to quantify symptom redundancy, the work by Gauld et al. (2021b) inventories all the different symptoms of sleep disorders from the clinical perspective of sleep medicine. Thus, this commentary shows the importance of a clinically informed split and lump of symptoms extracted from classifications. While specialized expertise may have led to an over-interpretation of the symptoms depicted by the criteria, the extraction from the whole DSM would have benefited from adding a specialist from each chapter to make them consistent with clinical practice.

## Note

1
https://osf.io/f3s4h
